# Supplementary material for: Evaluation of the effect of a communication skills course on medical students’ attitude towards patient-centered care: a prospective study
Source: PeerJ. 2024 Dec 9;12:e18676. doi: 10.7717/peerj.18676 (PMC11636530; doi:10.7717/peerj.18676)
Supplement: Supplemental Information 2 [file peerj-12-18676-s002.pdf]

PPOS communication skills questionnaire (Yr4-F)

Fill in a X for each item/question in the appropriate box of agreement

	ITEM		Strongly agree	Somewh at agree	Agree	Disagree	Somewhat disagree	Strongly disagree
1	The doctor is the one who should decide what gets talked about during a visit	PPOS01						
2	Although health care is less personal these days, this is a small price to pay for medical advances.	PPOS02						
3	The most important part of the standard medical visit is the physical examination.	PPOS03						
4	It is often best for patients if they do not have a full explanation of their medical condition.	PPOS04						
5	Patients should rely on their doctors’ knowledge and not try to find out about their conditions on their own.	PPOS05						
6	When doctors ask a lot of questions about a patient's background, they are prying too much into personal matters	PPOS06						
7	If doctors are truly good at diagnosis and treatment, the way they relate to patients is not that important.	PPOS07						
8	Many patients continue asking questions even though they are not learning anything new.	PPOS08						
9	Patients should be treated as if they were partners with the doctor, equal in power and status.	PPOS09						
10	Patients generally want reassurance rather than information about their health.	PPOS10						
11	If a doctor's primary tools are being open and warm, the doctor will not have a lot of success.	PPOS11						
12	When patients disagree with their doctor, this is a sign that the doctor does not have the patient's respect and trust.	PPOS12						
13	A treatment plan cannot succeed if it conflicts with a patient's lifestyle or values.	PPOS13						
14	Most patients want to get in and out of the doctor's office as quickly as possible	PPOS14						
15	The patient must always be aware that the doctor is in charge.	PPOS15						
16	It is not that important to know a patient's culture and background in order to treat the person's illness	PPOS16						
17	Humour is a major ingredient in the doctor's treatment of the patient.	PPOS17						
18	When patients look up medical information on their own, this usually confuses more than it helps.	PPOS18						
